# Supplementary material for: Single Cell Transcriptomic and Chromatin Profiles Suggest Layer Vb Is the Only Layer With Shared Excitatory Cell Types in the Medial and Lateral Entorhinal Cortex
Source: Front Neural Circuits. 2022 Jan 26;15:806154. doi: 10.3389/fncir.2021.806154 (PMC8826650; doi:10.3389/fncir.2021.806154)
Supplement: Supplementary file 14 [file Data_Sheet_1.ZIP › Exc_vs_Inh_Motifs/homerResults/motif2.similar.html]

motif2

## Information for motif2

T
G
A
C
G
T
A
C
C
G
T
A
A
C
G
T
T
G
C
A
C
G
A
T
A
C
T
G
A
C
T
G
G
A
C
T
G
T
C
A
  
Reverse Opposite:  

C
A
G
T
C
T
G
A
T
G
A
C
G
T
A
C
C
G
T
A
A
C
G
T
T
G
C
A
C
G
A
T
A
C
T
G
A
C
T
G
  

|  |  |
| --- | --- |
| p-value: | 1e-1307 |
| log p-value: | -3.010e+03 |
| Information Content per bp: | 1.630 |
| Number of Target Sequences with motif | 7761.0 |
| Percentage of Target Sequences with motif | 18.40% |
| Number of Background Sequences with motif | 760.3 |
| Percentage of Background Sequences with motif | 6.99% |
| Average Position of motif in Targets | 288.5 +/- 218.3bp |
| Average Position of motif in Background | 313.6 +/- 267.2bp |
| Strand Bias (log2 ratio + to - strand density) | 0.0 |
| Multiplicity (# of sites on avg that occur together) | 1.11 |
| Motif File: | file (matrix) reverse opposite |

### Similar de novo motifs found

|  |  |  |  |  |  |  |  |
| --- | --- | --- | --- | --- | --- | --- | --- |
| Rank | Match Score | Redundant Motif | P-value | log P-value | % of Targets | % of Background | Motif file |
| 1 | 0.936 | C T G A T G A C A G T C C G T A A C G T G T C A A C G T C T A G | 1e-937 | -2159.792803 | 16.42% | 6.98% | motif file (matrix) |
| 2 | 0.709 | C T G A A G T C G T A C C G T A C G A T A T G C G C A T A T C G C G A T G T A C | 1e-485 | -1118.895546 | 38.43% | 27.79% | motif file (matrix) |
